# The Convergent Validity of the electronic Frailty Index (eFI) with the Clinical Frailty Scale (CFS)

**DOI:** 10.3390/geriatrics5040088

**Published:** 2020-11-09

**Authors:** Antoinette Broad, Ben Carter, Sara Mckelvie, Jonathan Hewitt

**Affiliations:** 1 Community Services, Oxford Health NHS Foundation Trust, Oxford OX3 7JX, UK; antoinette.broad@oxfordhealth.nhs.uk; 2 Department of Biostatistics and Health Informatics, Institute of Psychiatry, Psychology & Neuroscience, King’s College London, London SE5 8AF, UK; ben.carter@kcl.ac.uk; 3 Emergency Medical Unit, Oxford Health NHS Foundation Trust, Oxford OX3 7JX, UK; sara.mckelvie@oxfordhealth.nhs.uk or; 4 Primary Care Research Group, Faculty of Medicine, University of Southampton, Southampton SO17 1BJ, UK; 5 Division of Population Medicine, Cardiff University, Penarth CF64 2XX, UK

**Keywords:** clinical Frailty Scale, electronic Frailty Index, community

## Abstract

**Background:** Different scales are being used to measure frailty. This study examined the convergent validity of the electronic Frailty Index (eFI) with the Clinical Frailty Scale (CFS). **Method:** The cross-sectional study recruited patients from three regional community nursing teams in the South East of England. The CFS was rated at recruitment, and the eFI was extracted from electronic health records (EHRs). A McNemar test of paired data was used to compare discordant pairs between the eFI and the CFS, and an exact McNemar Odds Ratio (OR) was calculated. **Findings:** Of 265 eligible patients consented, 150 (57%) were female, with a mean age of 85.6 years (SD = 7.8), and 78% were 80 years and older. Using the CFS, 68% were estimated to be moderate to severely frail, compared to 91% using the eFI. The eFI recorded a greater degree of frailty than the CFS (OR = 5.43, 95%CI 3.05 to 10.40; *p* < 0.001). This increased to 7.8 times more likely in men, and 9.5 times in those aged over 80 years. **Conclusions:** This study found that the eFI overestimates the frailty status of community dwelling older people. Overestimating frailty may impact on the demand of resources required for further management and treatment of those identified as being frail.

## 1. Introduction

Frailty has been defined as a state of vulnerability to adverse outcomes, as a consequence of cumulative decline in physiological systems and homeostatic reserve over the course of an individual’s lifetime [1]. Frailty is a distinct syndrome independent of disease [2].

With populations worldwide ageing rapidly frailty is high on everyone’s agenda, although the concept is well recognised, an international consensus to define frailty has yet to be reached [3]. In the absence of such a gold standard, many tools have been developed [4]. A standard measurement tool would provide a consistent recognition of frailty [5].

Due to age-related changes, frailty is diagnosed more often in older people [5]. Early identification of frailty can decrease the burden of disease, maintain independence longer and improve quality of life [6]. In response to this increasing evidence, NHS England published a new NHS long term plan [7] which calls for more proactive approaches to targeting interventions appropriately whilst contractually obliging General Practitioners (GPs) to identify patients over the age of 65 with moderate to severe frailty [8]. One frailty measure, the electronic Frailty Index (eFI) can be calculated by GPs using existing software and has become the GPs’ preferred frailty tool [9]. The Clinical Frailty Scale (CFS) is another validated measure of frailty based on clinical presentation. It is commonly used in clinical practice to diagnose frailty due to its simplicity [10]. It is essential that frailty is reproducible across different instruments used to identify it (convergent validity).

The study aimed to examine the convergent validity of the electronic Frailty Index (eFI) [11] with the 9- point Clinical Frailty Scale (CFS) [12] on community dwelling older people admitted onto a community nursing caseload.

## 2. Materials and Methods

### 2.1. Study Design

An observational design across three sites in the UK. The study was conducted in December 2018. As data used were collected as part of routine care, the study was deemed a service evaluation and was registered locally.

### 2.2. Participants

Community dwelling people (65 years or over) accepted on to three separate community nursing caseloads during one week in December 2018 were eligible to participate. Selected community nursing caseloads were determined by the following factors: geographical area, number of GP practices and location; city, town or village. Participants excluded from the study were those aged below 65 years.

### 2.3. Measures

The eFI is a cumulative deficit model that calculates frailty by retrieving patient information recorded from the electronic health record (EHR) [11]. It includes 36 deficit variables derived from diseases, symptoms and clinical signs recorded on the EHR. It can be calculated automatically on the EHR without the oversight of a clinician. Supplementary Table S1 shows the 36 deficits. It includes physical limitations including requirement of care, activity, mobility and transfer problems. It divides the total number of deficits present by the total possible creating a score between 0-1. The deficits each have a binary score of 1 if present and 0 when absent. eFI = Sum of deficits/36 (total number of deficits). The score is categorised accordingly into levels of severity, 0–0.12 = fit; >0.12–0.24 = mild frailty; >0.24–0.36 = moderate frailty and above 0.36 = severely frail [11].

The nine category Clinical Frailty Scale (CFS), is a validated measure of frailty based on clinical presentation. The assessor judges the level of frailty based on clinical findings and includes physical disability, cognition and co morbidity. It scores between one (very fit) to nine (terminally ill with a life expectancy of <6 months) [12]. Each point on the scale corresponds with a picture and written description to assist the clinician to grade the frailty score, a score = or >5 is frail [13]. Supplementary Figure S1 demonstrates the CFS.

### 2.4. Scoring Frailty

#### 2.4.1. eFI

Trained senior community nurses extracted the participants personal demographic data from community nursing teams’ EHR then accessed the participants eFI scores from the GP practice EHR.

#### 2.4.2. CFS

An assessment of frailty using the CFS was completed in the participants’ home during routine visits by community nurses within one week of their eFI screening. Training on how to complete the CFS was delivered by the study team. Nurses using the CFS were blinded to the recorded eFI score and classification. Participants were categorised as fit/mild or moderate/severely frail based on their reported scores.

The CFS and eFI were both dichotomised into two comparable scores of fit/mildly frail (CFS very fit, to mildly frail; eFI fit, to mildly frail), and moderately/severely frail (CFS moderately frail to terminally frail; eFI moderate to severely frail).

### 2.5. Statistical Analysis

Descriptive statistics were used to describe the population demographics and the distribution of frailty. Both the eFI and CFS were categorised into fit/mild, and moderate/severe. Categorisation of CFS followed the literature and 1–5 was compared to CFS 6–9, [14,15]; eFI was less common reported, and we mapped across a consistent threshold for this tool.

The McNemar test was used to test the association between the rating of the two frailty instruments: the eFI and the CFS [16]. The odds ratio (OR) and 95% confidence interval of over estimating frailty was calculated using the discordant pairs (e.g., comparing those more frail in the eFI (but not the CFS), was compared with those more frail in the CFS (but not the eFI)).

A subgroup analysis included patients aged over 80 years, as well as by gender. All analyses were carried out using Stata version 15.0.

## 3. Results

A total of 365 patients were recorded on the caseload, of which 327 met the eligibility criteria, 62 were removed due to incomplete data. Of those included, 150 (57%) were female, with a mean age of 85.6 years (SD = 7.8), and 78% were 80 years and older. Frailty prevalence estimates of moderate/severe were 91% (eFI), and 68% (CFS). (Table 1). There was a higher proportion of female patients in the moderate/severely frail group, but similar proportions of men and women in the fit/mildly frail group. Association between the frailty instruments was assessed by discordant pair analysis (Table 2).

Looking at the discordant pairs there were 76 patients seen by a clinician and found to have fit/mild frailty via the CFS but recorded moderate/severe frailty using the eFI, and inversely 14 patients scored moderate/severe with the CFS but as fit/mildly frail with the eFI. There was very strong evidence of an association of difference in the rate of discordant pairs (*p* < 0.001), and indication that the eFI is recorded with a greater degree of frailty. A patient being scored as more severely frail using the eFI compared to the CFS exhibited an OR = 5.43, (95%CI 3.05 to 10.40; *p* < 0.001).

Subgroup analysis estimated that this over scoring was more extreme in patients who were over 80 years old (*p* < 0.001), and in men (*p* < 0.001) (Table 3).

## 4. Discussion

This study found no evidence of convergent validity and the findings suggest that the eFI overestimates frailty among community dwelling older people. This overestimation increased for people greater than 80 years and for men. These findings conflict with recent studies that support the convergent validity of the two instruments [11,17] and endorse the eFI as a valid case finding tool to identify moderate/severely frail patients who may benefit from targeted interventions [11,18,19].

One explanation for the heterogeneity between scores might be that the two frailty instruments are underpinned by two very different theoretical frameworks. The CFS has been used widely (most recently during the COVID-19 pandemic) and has clinical judgement and has good face validity, it requires clinicians to undertake assessments of their patients’ comorbidities in real time [20]. Time to complete the CFS assessment and staff training are two main factors that limit the use of the CFS in practice [21]. In contrast the eFI is quick and requires minimal resources to complete; an accumulative deficit tool can be generated automatically on the GP system [11]. Utilising GP records for collecting patient data to assess frailty has been hailed as the gold standard [22]. The eFI is reliant upon clinicians applying clinical judgement and recording patient data accurately, and poor recording on the EHR can lead to absences of deficits or deficits can remain, even for temporary conditions [18]. Deficits no longer relevant to the patient’s current health state can lead to the eFI score being a biased estimate, potentially resulting in the reporting of the patient’s poorest estimate of frailty.

A second explanation for the divergent scores may be due to the population as housebound populations are often frail [23] so the findings from this study may not be comparable to the other studies that targeted total populations, their findings reported much lower levels of frailty [11]. Participants in the other studies that supported the convergent validity of the eFI and CFS were also less frail and younger than this study population [11,17]. This could offer an explanation as to why the scores were so different in that the eFI exhibited the worst status of the patient’s frailty and the convergent validity of the eFI and CFS may only be true for a younger, fit/mildly frail population. This study sample size and targeted population may limit the generalisability of the study findings.

## 5. Conclusions

The eFI has been recommended as a screening tool for frailty in primary care. This study suggests that the CFS offers current utility and the eFI overestimates the degree of frailty among housebound, older people, so use of the eFI may deplete health resources by directing services to people who do not require them. Thus, CFS should be preferred over the eFI. We recommend replicating this study on a larger scale to explore the above findings in more detail and to investigate the eFI as a case finding tool.

By having a greater understanding of frailty, clinicians can identify those at greatest risk of adverse health outcomes. Assessing frailty in older people over 65 years is now mandated in primary care in England [8]. The eFI is a popular choice due to its accessibility and ease of use but it may overestimate a patient’s true frailty status.

## Figures and Tables

**Table 1:** Demographic characteristics of the study population.

Category of frailty	eFI		CFS	
Fit/Mild	Moderate/Severe	Fit/Mild	Moderate/Severe
Total patients	23 (9%)	242 (91%)	85 (32%)	180 (68%)
Age (mean)	80.7	86	84.8	85.95
Male	10 (43%)	105 (43%)	43 (51%)	72 (40%)
Female	13 (57%)	137 (57%)	42 (49%)	108 (60%)

**Table 2:** Association between the electronic Frailty Index (eFI) and the Clinical Frailty Scale (CFS).

		CFS		
**eFI**	**Category of frailty**	Fit to Mild	Moderate to Severe	Total
	Fit to Mild	9	14	23
	Moderate to severe	76	166	242
	Total	85	180	265

**Table 3:** Subgroup of electronic Frailty Index (eFI) and the Clinical Frailty Scale (CFS) scores by age.

≤80 years	CFS		
eFI	Mild/fit	Moderate/severe	Total
Mild/fit	4 (a)	8 (b)	12 (a + b)
Moderate/severe	19 (c)	39 (d)	58 (c + d)
total	23 (a + c)	47 (b + d)	70
**>80 years**	**CFS**		
eFI	Mild/fit	Moderate/severe	Total
Mild/fit	5 (a)	6 (b)	11 (a + b)
Moderate/severe	57 (c)	127 (d)	184 (c + d)
total	62 (a + c)	133 (b + d)	195

## Supplementary Materials

The following are available online at https://www.mdpi.com/2308-3417/5/4/88/s1, Figure S1: Clinical Frailty Scale (CFS), Table S1: eFI list of 36 health deficits.
