# Supplementary material for: The Convergent Validity of the electronic Frailty Index (eFI) with the Clinical Frailty Scale (CFS)
Source: Geriatrics (Basel). 2020 Nov 9;5(4):88. doi: 10.3390/geriatrics5040088 (PMC7709678; doi:10.3390/geriatrics5040088)
Supplement: Supplementary file 1 [file geriatrics-05-00088-s001.pdf]

Supplementary files

Activity limitation	Hearing impairment	Polypharmacy
Anaemia and haematinic deficiency	Heart failure	Requirement for care
Arthritis	Heart valve disease	Respiratory disease
Atrial fibrillation	Housebound	Skin ulcer
Cerebrovascular disease	Hypertension	Sleep disturbance
Chronic kidney disease	Hypotension/syncope	Social vulnerability
Diabetes	Memory and cognitive problems	Thyroid disease
Dizziness	Mobility and transfer problems	Urinary incontinence
Dyspnoea	Osteoporosis	Urinary system disease
Falls	Parkinsonism and tremor	Visual impairment
Foot problems	Peptic ulcer	Weight loss and anorexia
Fragility fracture	Peripheral vascular disease	Ischaemic heart disease

Table 1. eFI list of 36 health deficits [11]

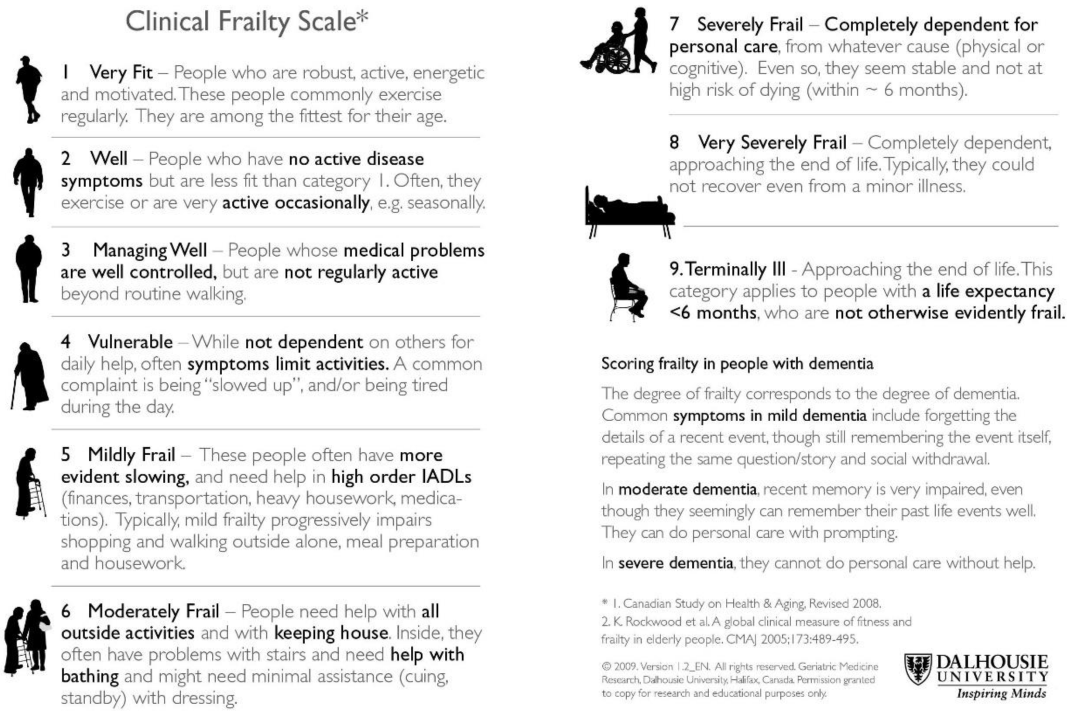

Figure 1. Clinical frailty scale (CFS) [12]
